# Associations with antibiotic prescribing for acute exacerbation of COPD in primary care: secondary analysis of a randomised controlled trial

**DOI:** 10.3399/BJGP.2020.0823

**Published:** 2021-03-09

**Authors:** David Gillespie, Christopher C Butler, Janine Bates, Kerenza Hood, Hasse Melbye, Rhiannon Phillips, Helen Stanton, Mohammed Fasihul Alam, Jochen WL Cals, Ann Cochrane, Nigel Kirby, Carl Llor, Rachel Lowe, Gurudutt Naik, Evgenia Riga, Bernadette Sewell, Emma Thomas-Jones, Patrick White, Nick A Francis

**Affiliations:** Nuffield Department of Primary Care Health Sciences, University of Oxford, Oxford, UK; Centre for Trials Research, School of Medicine, College of Biomedical and Life Sciences, Cardiff University, Cardiff, UK.; Nuffield Department of Primary Care Health Sciences, University of Oxford, Oxford, UK.; Centre for Trials Research, School of Medicine, College of Biomedical and Life Sciences, Cardiff University, Cardiff, UK.; Centre for Trials Research, School of Medicine, College of Biomedical and Life Sciences, Cardiff University, Cardiff, UK.; General Practice Research Unit, Department of Community Medicine, UIT the Arctic University of Norway, Tromsø, Norway.; Cardiff School of Sport and Health Science, Cardiff Metropolitan University, Cardiff, UK.; Centre for Trials Research, School of Medicine, College of Biomedical and Life Sciences, Cardiff University, Cardiff, UK.; Department of Public Health, College of Health Sciences, QU-Health, Qatar University, Doha, Qatar.; Department of Family Medicine, School for Public Health and Primary Care, Medicine and Life Sciences, Maastricht University, Maastricht, the Netherlands.; York Trials Unit, Department of Health Sciences, University of York, York, UK.; Centre for Trials Research, School of Medicine, College of Biomedical and Life Sciences, Cardiff University, Cardiff, UK.; Research Unit for General Practice, Department of Public Health, University of Copenhagen, Copenhagen, Denmark; University Institute in Primary Care Research Jordi Gol, Via Roma Health Centre, Barcelona, Spain.; Centre for Trials Research, School of Medicine, College of Biomedical and Life Sciences, Cardiff University, Cardiff, UK.; Division of Population Medicine, School of Medicine, Cardiff University, Cardiff, UK.; Department of Psychiatry, Medical Sciences Division, University of Oxford, Oxford, UK.; Swansea Centre for Health Economics, Swansea University, Swansea, UK.; Centre for Trials Research, School of Medicine, College of Biomedical and Life Sciences, Cardiff University, Cardiff, UK.; School of Population Health and Environmental Sciences, King’s College London, London, UK.; Primary Care, Population Sciences and Medical Education, University of Southampton, Aldermoor Health Centre, Southampton, UK.

**Keywords:** antibiotics, COPD, C-reactive protein, primary care, randomised controlled trial

## Abstract

**Background:**

C-reactive protein (CRP) point-of-care testing can reduce antibiotic use in patients with acute exacerbation of chronic obstructive pulmonary disease (AECOPD) in primary care, without compromising patient care. Further safe reductions may be possible.

**Aim:**

To investigate the associations between presenting features and antibiotic prescribing in patients with AECOPD in primary care.

**Design and setting:**

Secondary analysis of a randomised controlled trial of participants presenting with AECOPD in primary care (the PACE trial).

**Method:**

Clinicians collected participants’ demographic features, comorbid illnesses, clinical signs, and symptoms. Antibiotic prescribing decisions were made after participants were randomised to receive a point-of-care CRP measurement or usual care. Multivariable regression models were fitted to explore the association between patient and clinical features and antibiotic prescribing, and extended to further explore any interactions with CRP measurement category (CRP not measured, CRP <20 mg/l, or CRP ≥20 mg/l).

**Results:**

A total of 649 participants from 86 general practices across England and Wales were included. Odds of antibiotic prescribing were higher in the presence of clinician-recorded crackles (adjusted odds ratio [AOR] = 5.22, 95% confidence interval [CI] = 3.24 to 8.41), wheeze (AOR = 1.64, 95% CI = 1.07 to 2.52), diminished vesicular breathing (AOR = 2.95, 95% CI = 1.70 to 5.10), or clinician-reported evidence of consolidation (AOR = 34.40, 95% CI = 2.84 to 417.27). Increased age was associated with lower odds of antibiotic prescribing (AOR per additional year increase = 0.98, 95% CI = 0.95 to 1.00), as was the presence of heart failure (AOR = 0.32, 95% CI = 0.12 to 0.85).

**Conclusion:**

Several demographic features and clinical signs and symptoms are associated with antibiotic prescribing in AECOPD. Diagnostic and prognostic value of these features may help identify further safe reductions.

## INTRODUCTION

Chronic obstructive pulmonary disease (COPD) affects around 2% of the UK population, and is the third leading cause of death globally.^1^^–^^3^ More than 70% of patients presenting with acute exacerbations of COPD (AECOPD) in primary care are prescribed an antibiotic,^4^ despite bacterial pathogens only being detectable in 20%–50% of exacerbations.^5^^–^^7^ Antibiotic use for AECOPD accounts for 7.5% of all primary care antibiotic prescriptions.^8^

Overuse of antibiotics contributes to the development of antimicrobial resistance, exposes patients to the risk of unnecessary side effects, wastes money, and undermines self-care.^9^ There is, therefore, a need for antimicrobial stewardship initiatives to target the prescribing of antibiotics for patients with AECOPD in primary care.

The PACE trial demonstrated that a management strategy involving the use of C-reactive protein (CRP) point-of-care testing (POCT) for patients with AECOPD in primary care can lead to a reduction in antibiotic use without any evidence of patient harm.^10^ In this randomised controlled trial, antibiotics were used by 77% of patients in the usual-care group compared with 57% in the CRP-POCT group (a relative difference of 26%), while potential bacterial pathogens were isolated in the sputum of only 44% of participants. This suggests that there may be potential for further reductions in antibiotic prescribing, and it is therefore important to understand the determinants of antibiotic prescribing for this condition.

The aim of this study was to examine the presenting features associated with antibiotic prescribing decisions for AECOPD in primary care, and to explore if these were different when clinicians had access to a CRP measurement (available for patients randomised to the intervention arm of the trial), and whether the CRP value was elevated (≥20 mg/l) or not.

**Table:** How this fits in

Overuse of antibiotics contributes to antimicrobial resistance, unnecessarily exposes patients to side effects, and undermines self-care. A recent randomised controlled trial demonstrated that, when clinical management is supplemented with a C-reactive protein (CRP) point-of-care test, antibiotics can be safely reduced in patients presenting in primary care with an acute exacerbation of chronic obstructive pulmonary disease (AECOPD). Further analysis, in the present study, found several demographic and clinical features associated with the prescribing of antibiotics to patients presenting with AECOPD in UK primary care, independent of the CRP test result. Studying the diagnostic and prognostic value of these features is warranted to understand how to safely reduce antibiotic use in this population.

## METHOD

### Study design

This was a secondary analysis of an open, multi-site, parallel-group, individually randomised controlled trial that evaluated the effectiveness of a CRP-POCT management strategy for patients with an AECOPD in UK primary care (the PACE trial). Target recruitment was 650 participants. The protocol and findings for the original study are reported elsewhere.^10^^–^^12^

### Participants and setting

Participants aged ≥40 years with a clinically recorded diagnosis of COPD (with or without spirometry confirmation) who presented with an acute exacerbation for 1–21 days were recruited into the trial from general practices across England and Wales. Full inclusion and exclusion criteria have been described previously.^11^

### Procedures

After patients gave informed consent, their baseline data were collected. Participants were then randomly allocated using remote online computer randomisation (ratio 1:1) either to management via usual care (no CRP-POCT) or to CRP-POCT in addition to usual care.

All general practices (*n*= 86) were provided with a POCT device and all associated materials, information on current best practice for managing AECOPD, with no other specific guidance given to clinicians with regards to the management of their patients. Participants allocated to the usual-care arm were managed without the use of a CRP-POCT measurement. Those allocated to the CRP-POCT arm had a CRP measurement taken using a POCT desktop machine, to help guide initial antibiotic prescribing decisions. Clinicians received guidance and training on how to use the device and interpret the result. The guidance indicated that antibiotics were unlikely to be beneficial and should usually not be prescribed for patients with a CRP <20 mg/l; that antibiotics may be beneficial, especially if purulent sputum is present, for patients with a CRP 20–40 mg/l; and that antibiotics are likely to be beneficial and should usually be prescribed (unless a patient is assessed as being at low risk of complications) for patients with a CRP >40 mg/l. The CRP cut-offs were based on data from a placebo-controlled trial of antibiotics for patients with acute exacerbations of mild-to-moderate COPD.^13^

Clinicians recorded participants’ demographic details (age and sex), their medical history (presence of comorbidities and COPD stage according to Global Initiative for Chronic Obstructive Lung Disease [GOLD] criteria), and clinical features pertaining to their exacerbation: number of days experiencing symptoms, temperature, pulse rate, oxygen saturation, ability to complete a full sentence, tachypnoea, presence and number of Anthonisen symptoms (increased shortness of breath, increased sputum volume, and/or increased sputum purulence),^14^ presence of crackles, wheeze, diminished vesicular sounds, or evidence of consolidation on auscultation of the lungs. Clinicians recorded the colour of the sputum sample using a BronkoTest chart.^15^ Where a sputum sample could not be obtained, participants estimated their current sputum colour using the chart. Participants were also asked about their smoking status (nonsmoker, current smoker, or ex-smoker) during 1-week follow-up assessments.

Antibiotic prescribing and other management decisions were made and recorded after receipt of the test result for those allocated to the CRP-POCT arm.

### Statistical methods

Descriptive statistics were reported as frequencies and percentages or means and standard deviations, as appropriate.

To investigate the association between patient and clinical features and antibiotic prescribing, multilevel multivariable logistic regression models were fitted, accounting for any clustering of participants in practices. Each explanatory variable (those described in the Procedures section) was included in separate regression models. Sputum colour was ranked 1 (lightest colour) to 5 (darkest colour). Continuous variables were grand-mean-centred and included as linear effects following the inspection of model parsimony, when comparing linear terms with restricted cubic splines with both five and three knots, using the Akaike information criterion (see Supplementary Table S1 for details). Each model was adjusted for CRP measurement (defined as no measurement taken, CRP <20 mg/l, and CRP ≥20 mg/l) in addition to increased sputum purulence. Sputum purulence was adjusted for as a potential confounder as it was an exacerbation feature specifically mentioned in the guidance on interpreting the CRP measurement. The differential association between explanatory variables and antibiotic prescribing by CRP measurement was explored by extending models to include CRP measurement interacted with explanatory variables.

The proportion of the total variance, for explanatory variable, that was attributable to differences across practices was expressed by estimating the intra-cluster correlation coefficient (ICC), with the π^2^/3 estimator used where considering a binary response. These were calculated to indicate practice (as a proxy for prescriber) variation in the reporting of these features.

Statistical analyses were conducted using Stata (version 16.0).

## RESULTS

### Participant flow

**Figure 1.**
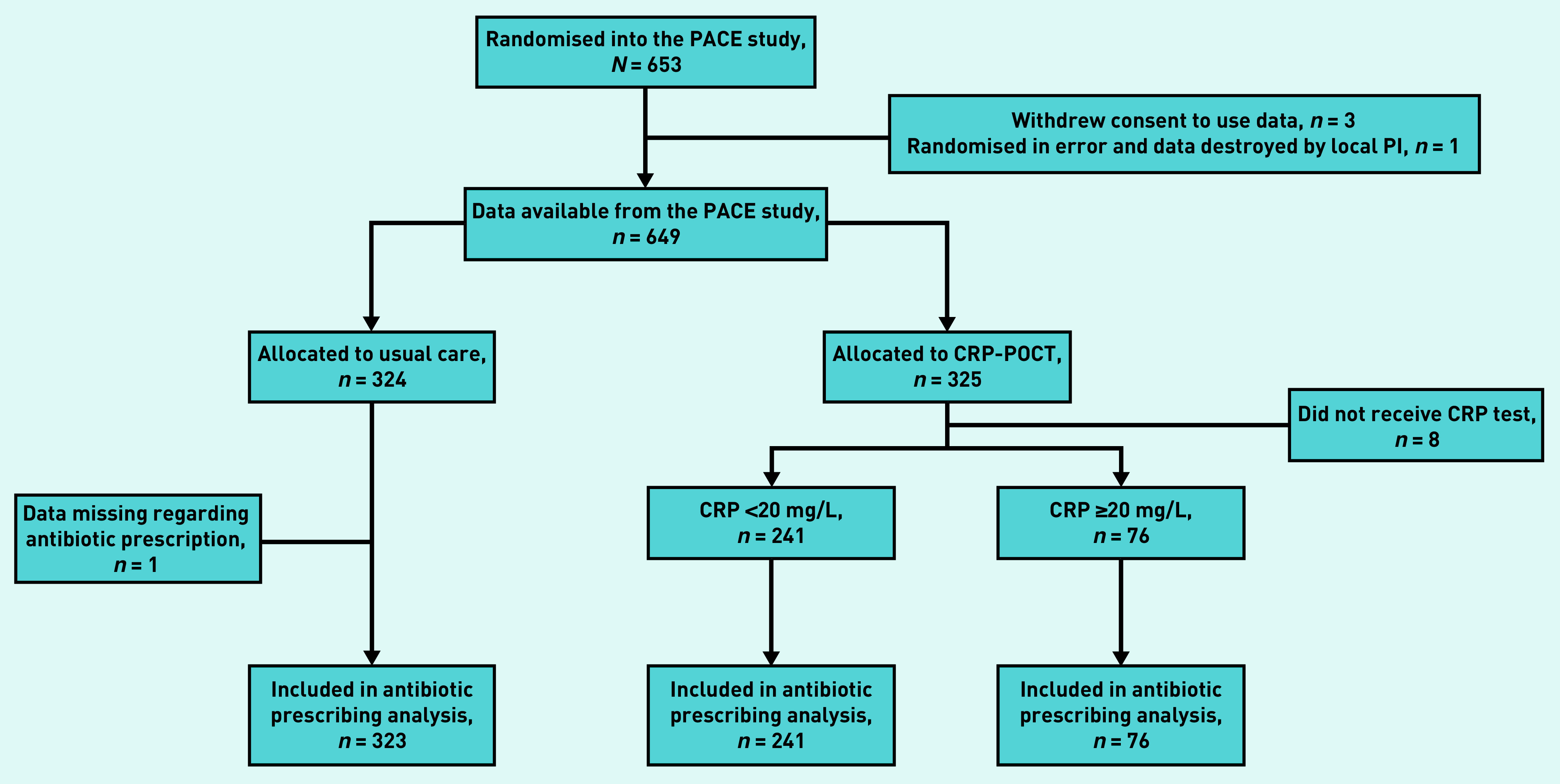
***Participant flow diagram.*** ***CRP = C-reactive protein. PI = principal investigator. POCT = point-of-care test.***

The PACE trial consented and randomised 653 participants from 86 general practices across England and Wales. Three participants withdrew their permission for their data to be used and one was randomised in error, leaving 649 participants: 324 were allocated to usual care and 325 to CRP-POCT. CRPPOCT data were not available for eight participants, leaving 241 allocated to CRPPOCT with a CRP value <20 mg/l, and 76 allocated to CRP-POCT with a CRP value ≥20 mg/l (Figure 1). See Supplementary Table S2 for details of descriptive statistics overall, by CRP measurement, and by antibiotic prescription receipt at the index consultation.

### Numbers analysed

One participant (CRP not measured) did not have data available regarding antibiotic prescribing decisions at the index consultation. Data availability varied for each of the candidate variables, and numbers of participants for each are given in Table 1. Antibiotics were prescribed at the index consultation to 225 (69.7%) participants in whom CRP was not measured, 79 (32.8%) with CRP <20 mg/l, and 68 (89.5%) with CRP ≥20 mg/l (see Supplementary Table S2 for details).

**Table 1.** Associations between demographic features, comorbid illness, symptoms and signs, and antibiotic prescribing at the index consultation

**Variable^a^**	**Adjusted odds ratio^b^**	**95% CI**	***P*-value**
**Demographic features and comorbid illness**			

Age, years (*n* = 640)	0.98	0.95 to 1.00	0.035

**Sex (*n* = 640)**			
Male	Ref		
Female	1.17	0.77 to 1.78	0.472

Heart failure (*n* = 640)	0.32	0.12 to 0.85	0.022

Chronic heart disease (*n* = 640)	0.89	0.52 to 1.51	0.657

Diabetes (*n* = 640)	1.43	0.81 to 2.50	0.215

Chronic kidney disease (*n* = 640)	1.76	0.81 to 3.81	0.151

Hypertension (*n* = 640)	1.02	0.66 to 1.56	0.934

Other chronic disease (*n* = 581)	0.80	0.48 to 1.32	0.379

At least one comorbid illness (*n* = 625)	0.85	0.53 to 1.34	0.479

**Smoking status (*n* = 551)**			
Non-smoker	Ref		
Current smoker	1.14	0.45 to 2.88	0.777
Ex-smoker	1.08	0.45 to 2.59	0.867

**COPD severity (*n* = 551)**			
GOLD stage 1 (mild)	Ref		
GOLD stage 2 (moderate)	1.52	0.83 to 2.81	0.179
GOLD stage 3 (severe)	1.62	0.81 to 3.27	0.176
GOLD stage 4 (very severe)	1.15	0.43 to 3.10	0.782

**Symptoms and signs**			

Days with symptoms (per day) (*n* = 640)	0.98	0.94 to 1.02	0.235

Increased breathlessness (*n* = 640)	1.72	0.86 to 3.41	0.124

Increased sputum volume (*n* = 640)	1.40	0.85 to 2.31	0.181

**Sputum colour (*n* = 568)**			
1 (lightest)	Ref		
2	0.79	0.40 to 1.54	0.485
3	1.38	0.69 to 2.76	0.358
4	0.82	0.40 to 1.68	0.587
5 (darkest)	2.37	0.80 to 6.98	0.119

Crackles (*n* = 640)	5.22	3.24 to 8.41	<0.001

Wheeze (*n* = 640)	1.64	1.07 to 2.52	0.022

Diminished vesicular breathing (*n* = 638)	2.95	1.70 to 5.10	<0.001

Clinician-reported evidence of consolidation (*n* = 638)	34.40	2.84 to 417.27	0.005

Patient cannot complete a full sentence without stopping (*n* = 581)	1.30	0.46 to 3.66	0.623

Patient is tachypnoeic (*n* = 581)	1.30	0.70 to 2.43	0.405

Temperature (*n* = 639)	1.33	0.87 to 2.04	0.186

Pulse rate (*n* = 639)	1.01	0.99 to 1.03	0.250

Oxygen saturation (*n* = 637)	0.96	0.89 to 1.05	0.397

Patient has been prescribed antibiotics in the past 12 months (*n* = 597)	0.95	0.60 to 1.49	0.809

a n *-values indicate number of available data for each variable.*

b Model adjusts for C-reactive protein (CRP) measurement (CRP measurement not available, CRP <20 mg/l, and CRP ≥20 mg/l), the presence of sputum purulence, and the clustered nature of participants within practices. CI = confidence interval. COPD = chronic obstructive pulmonary disease. GOLD = Global Initiative for Chronic Obstructive Lung Disease.

### Demographic features and comorbid illness

Higher participant age was associated with lower odds of antibiotic prescribing (adjusted odds ratio [AOR] per additional year increase = 0.98, 95% confidence interval [CI] = 0.95 to 1.00, *P* = 0.035) (Table 1). The presence of heart failure was associated with lower odds of antibiotic prescribing (AOR = 0.32, 95% CI = 0.12 to 0.85, *P*= 0.022). There was no evidence that the association between any of the patient characteristics and antibiotics were different by CRP measurement (see Supplementary Table S3 for details).

Practice-level ICCs for demographic features and comorbid illness ranged from 0.02 (95% CI = 0.00 to 0.17) for age to 0.13 (95% CI = 0.05 to 0.28) for the presence of at least one comorbid illness (see Supplementary Figure S1 for details).

### Symptoms and signs

Clinician-reported chest auscultation findings of crackles (AOR = 5.22, 95% CI = 3.24 to 8.41, *P*<0.001), wheeze (AOR = 1.64, 95% CI = 1.07 to 2.52, *P*= 0.022), and diminished vesicular breathing (AOR = 2.95, 95% CI = 1.70 to 5.10, *P*<0.001), as well as clinician-reported evidence of consolidation (AOR = 34.40, 95% CI = 2.84 to 417.27, *P* = 0.005), were all associated with higher odds of antibiotic prescribing (Table 1). A greater number of participants in the high CRP group (59.2%) experienced crackles than did those in the low CRP group (44.0%) (see Supplementary Table S2).

ICCs for clinical features ranged from 0.02 (95% CI = 0.00 to 0.15) for pulse rate to 0.60 (95% CI = 0.32 to 0.81) for evidence of consolidation on auscultation of the lungs (see Supplementary Figure S2 for details).

**Figure 2.**
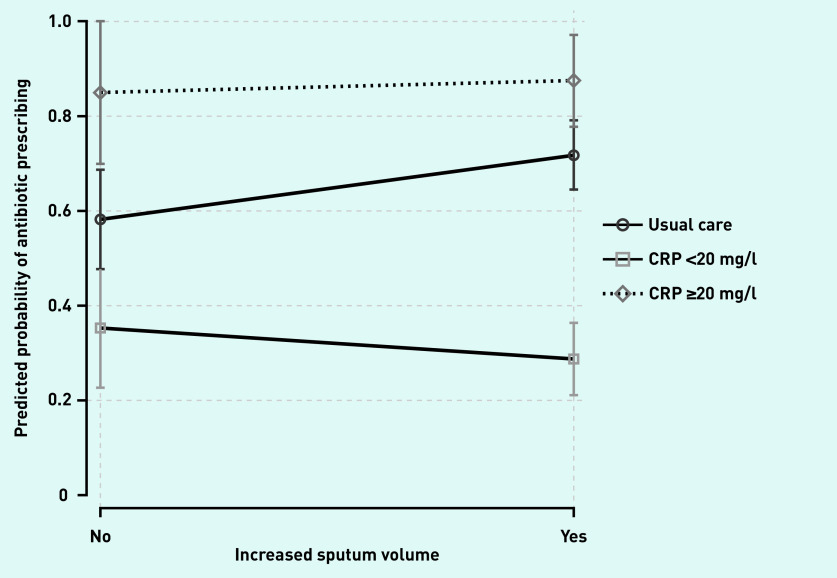
***Differential association between increased sputum volume and antibiotic prescribing by CRP measurement. Vertical lines indicate 95% CIs.*** ***CI = confidence interval. CRP = C-reactive protein.***

**Figure 3.**
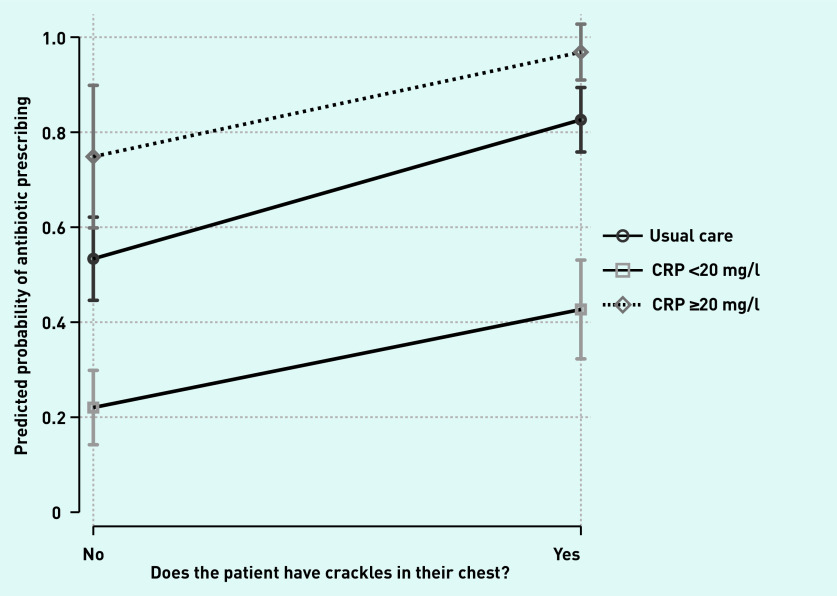
***Differential association between reporting crackles on auscultation and antibiotic prescribing by CRP measurement. Vertical lines indicate 95% CIs.*** ***CI = confidence interval. CRP = C-reactive protein.***

There was evidence to suggest a differential association between increased sputum volume and antibiotic prescribing by CRP measurement. Specifically, while an increase in sputum volume was associated with higher odds of antibiotic prescribing for participants in whom CRP was not measured (increased sputum volume main effect odds ratio [OR] = 2.18, 95% CI = 1.17 to 4.07; CRP <20 mg/l main effect = 0.30, 95% CI = 0.13 to 0.65; CRP ≥20 mg/l main effect: 5.88, 95% CI = 1.36 to 25.50) (see Supplementary Table S3 for details), there was minimal influence on antibiotic prescribing for those with CRP <20 mg/l (interaction between increased sputum volume and CRP <20 mg/l OR = 0.31, 95% CI = 0.12 to 0.80) (Figure 2). Evidence of consolidation was reported for 18 participants in total (seven in those for whom CRP was not measured, eight with CRP <20 mg/l, and three with CRP ≥20 mg/l). All but one of these participants (who had CRP <20 mg/l) were prescribed antibiotics at the index consultation (data not shown). The reporting of crackles was associated with the highest odds of antibiotic prescribing, and there was no evidence of a differential association by CRP measurement (Figure 3).

## DISCUSSION

### Summary

This study investigated antibiotic prescribing associations for patients with AECOPD in UK primary care. It found that lower age, presence of heart failure, and clinician-reported abnormal findings on examination of the lungs (crackles, wheeze, diminished vesicular breathing, and evidence of consolidation) were all associated with antibiotic prescribing at the index consultation after adjusting for CRP measurement category and the presence of increased purulent sputum.

Increased patient-reported sputum volume was associated with antibiotic prescribing when CRP was not measured, but considerably less so when it was measured. Reporting crackles on auscultation was the feature most strongly associated with antibiotic prescribing, and the magnitude of this association was large across all three CRP measurement groups.

### Strengths and limitations

These data were obtained from the largest trial of patients with AECOPD in UK primary care, covering 86 general practices across England and Wales. The trial benefitted from a representative sample of this patient population,^8^^,^^16^ and, with high data completion, most participants were retained for these analyses. Clinicians in the participating practices were trained in study procedures and data collection processes in accordance with a standardised protocol, and this minimised any biases arising from variable research practices.

This was a secondary analysis of a randomised controlled trial, and no formal power calculation was conducted for these particular analyses. Furthermore, the ICC estimates should be interpreted with some caution, as these were obtained from data arising from a randomised controlled trial, and the sources of variation may reflect on the type of person a clinician was willing to include in such a trial. In addition, the calculation of ICC values on the log-odds scale for binary variables, while not depending on cluster prevalence, may not directly translate to other studies. The ICC of 0.60 for clinician-reported evidence of consolidation likely reflects the variability in clinical assessment of this feature, as well as how rare it is in primary care. Finally, it is not possible to draw causal conclusions regarding the presenting features and their relationship to antibiotic prescribing.

The considerable practice variation in recording of clinical features suggests a high degree of subjectivity, as has been shown in previous studies.^17^^,^^18^ It is also not possible to rule out a relationship between clinical features and antibiotic prescribing being confounded by clinicians’ perceptions of the need for antibiotics, which have previously been shown to influence the recording of ‘objective’ features such as clinical findings and diagnosis.^19^

### Comparison with existing literature

Several of the study’s findings are consistent with previous studies on the determinants of antibiotic prescribing for acute cough/lower respiratory tract infection in primary care, including crackles, wheeze, and diminished breath sounds (and other abnormal auscultation findings).^20^^–^^23^

The finding that increasing age was associated with lower odds of antibiotic prescribing was unexpected and inconsistent with the study by Llor and colleagues.^4^ The current study differs in two key ways. First, it was a randomised controlled trial with several eligibility criteria, whereas the study by Llor and colleagues was an observational study, in which clinicians included all patients over a defined time period. Thus, the current study may have inadvertently excluded older participants who would more likely be prescribed antibiotics, despite there being no upper age limit. Second, the association between age and antibiotic prescribing in the current study was adjusted for CRP measurement and increased sputum purulence (none, <20 mg/l, or ≥20 mg/l), whereas the study by Llor and colleagues was adjusted for several variables in a multivariable analysis (sex, days with symptoms, several different types of symptoms, utilisation of CRP, clinician request for a chest X-ray, and patient demanding antibiotics).

The weak association between sputum volume and antibiotic prescribing may indicate that clinicians are less certain about an increase in sputum volume as an indicator of bacterial infection compared with other clinical features. This is in line with the GOLD statement^24^ that sputum purulence is the strongest predictor of bacterial infection among the Anthonisen criteria, and that sputum volume and increased dyspnoea should not be emphasised in the absence of purulence. This point of view is supported by Miravitlles and colleagues,^25^ who found that purulence was the only Anthonisen criterion independently predicting an unfavourable outcome in patients with AECOPD treated with placebo.

### Implications for research

Clinicians use a range of demographic and clinical features, including age and lung sounds, in their decision to prescribe antibiotics to patients presenting with AECOPD in UK primary care. In this study, the importance attributed to chest findings, and crackles in particular, in deciding on prescribing of antibiotics for AECOPD is not supported by a strong evidence base nor included in current guidelines. The emphasis on crackles by clinicians is probably related to the increased frequency found in pneumonia.^26^ However, crackles are commonly heard in COPD,^27^ and especially during exacerbations, related to worsened bronchial obstruction.^28^ In the present study, while the link between crackles and antibiotic prescribing was independent of CRP result, a greater number of participants in the high CRP group (59.2%) experienced crackles than did those in the low CRP group (44.0%), indicative of a relationship between crackles and more seriously unwell participants. Further investigation is required to determine the diagnostic and prognostic value of crackles and other chest sounds, and whether further safe reductions in antibiotic prescribing for AECOPD are possible.

## Data sharing

Data available from the authors on request.
